# Analysis of Domain-Swapped Oligomers Reveals Local Sequence Preferences and Structural Imprints at the Linker Regions and Swapped Interfaces

**DOI:** 10.1371/journal.pone.0039305

**Published:** 2012-07-27

**Authors:** Prashant Shingate, R. Sowdhamini

**Affiliations:** National Centre for Biological Sciences, UAS-GKVK Campus, Bangalore, India; Uni. of South Florida, United States of America

## Abstract

**Background:**

3D domain swapping is an oligomerization process in which structural elements get exchanged between subunits. This mechanism grasped interest of many researchers due to its association with neurodegenerative diseases like Alzheimer's disease, spongiform encephalopathy *etc*. Despite the biomedical relevance, very little is known about understanding this mechanism. The quest for ruling principles behind this curious phenomenon that could enable early prediction provided an impetus for our bioinformatics studies.

**Methodology:**

A novel method, HIDE, has been developed to find non-domain-swapped homologues and to identify hinge from domain-swapped oligomers. Non-domain-swapped homologues were identified from the protein structural databank for majority of the domain-swapped entries and hinge boundaries could be recognised automatically by means of successive superposition techniques. Different sequence and structural features in domain-swapped proteins and related proteins have also been analysed.

**Conclusions:**

The HIDE algorithm was able to identify hinge region in 83% cases. Sequence and structural analyses of hinge and interfaces reveal amino acid preferences and specific conformations of residues at hinge regions, while comparing the domain-swapped and non-domain-swapped states. Interactions differ significantly between regular dimeric interfaces and interface formed at the site of domain-swapped examples. Such preferences of residues, conformations and interactions could be of predictive value.

## Introduction

3D-domain swapping is a well-known but poorly understood mechanism of oligomer formation. This mechanism was first named by Eisenberg and coworkers after their structure of diphtheria toxin in 1994 [1]. In this mechanism, whole structural domains or small structural elements get exchanged between identical or similar chains, which lead to the formation of dimer or higher order oligomers. Before exchange of structural elements between these monomeric subunits, they undergo partial unfolding to form open conformers. These open conformers further undergo domain swapping at high concentrations.

There are different categories of domain-swapped oligomers present, depending on the existence of the monomeric form described by Eisenberg and coworkers [2]. First category of domain swapping is ‘*bonafide* domain swapping’ in which a given protein molecule is known to exist in monomeric as well as in domain-swapped form *e.g.*: cyanovirin-N monomer [3] and dimer [4]. Another is ‘quasi-domain swapping’, in which a given protein is known to exist in the oligomeric form, but its close homologue is known to exist in monomeric form e.g.: human cystatin C dimer [5] and chicken cystatin monomer [6]. In the third category, domain-swapped form is observed in a protein of given structure, but structural information of their monomers or monomeric homologues are not available; these are referred as ‘candidates for 3D domain swapping’. In ‘candidates for 3D domain swapping’, hinge boundaries are ambiguous due to the lack of structure of the monomeric form *e.g.*: phosphoenol pyruvate mutase dimer [7].

Domain swapping is also associated with amyloid formation, which ultimately leads to neurodegenerative diseases like Alzheimer's disease, spongiform encephalopathy and human cystatin-C [8].

**Figure 1:**
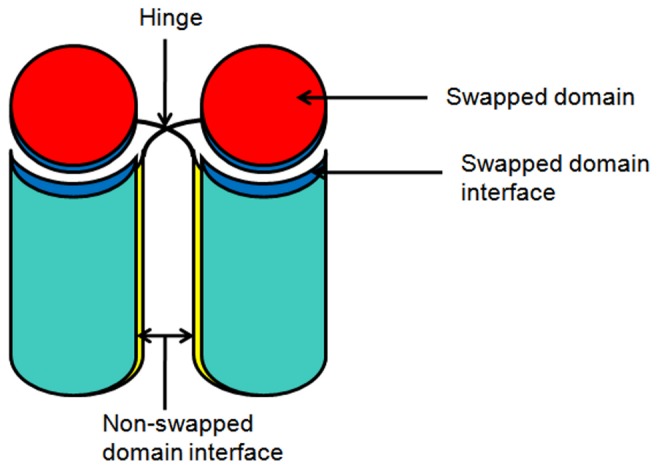
Domain-swapped oligomers with different regions marked.

Domain-swapped oligomers have few structural characteristics which differentiate them from other side-by-side oligomers. First obvious characteristic is the swapped region or domain. ‘Swapped domain’ (SD) is a structural element which gets exchanged in 3D-domain-swapped oligomerization (**Figure 1**). This swapped domain can be as small as a three residue β-strand [9] (as in mouse GITRL) or whole globular domain (as observed in diphtheria toxin [1]). Another important feature in domain swapping is the presence of a ‘hinge region’ (**Figure 1**). It is a linker connecting the swapped domain to the rest of the portion of protein and allows movement of swapped domain during the swapping process.

Swapped domain interface and non-swapped domain interfaces (**Figure 1**) forms after oligomerization between monomeric subunits. ‘Swapped domain interface’ (DSI) is intermolecular interface formed between swapped domain and rest of the portion of the protein. This interface is the energetically optimized interface during the course of evolution which gets utilized in the oligomeric form more than once [10]. This is the major advantage of domain-swapped oligomers over other oligomers. Mutation in this interface may induce domain-swapping in some proteins e.g.: NF-β p50 protein [11]. Eisenberg had shown that weakening of this interface is important for partial unfolding and thus for domain swapping [2]. While ‘non-swapped domain interface’ (NDSI) is a newly formed interface formed between monomeric subunits. Oligomerization through domain-swapping highly depends upon the strength of interactions within this interface.

3DSwap [12] (Khader et al., 2011) is an in-house knowledgebase for domain-swapped entries from PDB database. In this database, information about hinge boundaries and swapped domain regions has been added after extensive literature curation. Common ways to find important hinge residue is either by mutational studies, which is a laborious task, or using homologous non-domain-swapped form. A decision of the critical hinge residue boundaries for domain swapping and searching their non-domain-swapped homologues had remained quite time-consuming and manually driven during the curation process. AdiDos [13] is a hinge identification algorithm, reported in the literature, which requires non-domain-swapped form as an input from the user. Search of such non-domain-swapped form might again be a time-consuming task.

In this work, we have developed an algorithm, HIDE (**H**inge **IDE**ntification), which begins by searching for protein structure/s of the same molecule and its homologous proteins present in non-domain-swapped form in PDB automatically. To check the domain-swapped or non-domain-swapped status of these homologous protein structures, we implemented a new strategy, which employs the least square plane. The non-domain-swapped homologues, thus identified, were further used to identify hinge region using successive rigid-body superposition and structural alignment approach. This algorithm not only identifies hinge boundaries, but also marks residues critical for the domain-swapping event as evident from high root mean square deviation (r.m.s.d.) values.

Further, our group had earlier showed that whether a protein will be engaged in domain swapping or not can be predicted from mere sequence information. We also developed a server, 3DSwap-pred [14] which predicts 3D-domain swapping event from sequence data alone. However, knowledge about the exact regions involved in hinge and swapped regions and the structural features of the DSI and NDSI are still incomplete. Therefore, we have now analysed sequence specificity and (φ, ψ) angle distribution of hinge residues in the domain-swapped form and its equivalent region in its non-domain-swapped homologue. We find there are specific amino acid propensities and backbone torsion angles adopted at the hinge region of the domain-swapped proteins, in comparison to non-domain-swapped counterparts, which could be of predictive value.

## Materials and Methods

### Dataset

Domain-swapped entries were obtained from 3D-Swap database for our analysis. Redundant entries with more than 90% identity were filtered out using CD-hit tool [15] and a further filter was to use only structures with resolution better than 3.2 Å (listed in Table S1).

### Search for homologous proteins in PDB

Both BLAST [16] and DALI [17] tools were used, with default parameters, against the PDB database to search for non-domain-swapped homologues. Homologues showing more than 30% identity and E-value less than 10^−3^ were used as non-domain-swapped homologue for further hinge identification process. In case of multiple occurrence of the same entry as a homologue during sequence search, their average identity was considered. The homologues were assigned domain-swapped and non-domain-swapped status using the following algorithm.

### Assignment of oligomeric status of the homologues

The oligomeric status of all proteins were examined by consulting PISA [18] and PQS [19] (Protein Quaternary Structures) database. The proteins in the monomeric forms were straightaway marked as ‘non-domain-swapped’ structures. While oligomeric homologues were assigned domain-swapped or non-domain-swapped status using least square plane approach and then hinge identified through HIDE algorithm.

### Hinge Identification Algorithm (HIDE)

HIDE algorithm consists of two major steps as follows:

#### Step 1: Search of non-domain-swapped homologues

In the case of protein structural entries of dimers or higher order oligomers, all interchain residue distances were calculated and pairs with distance less than 7 Å were considered as interface residues. Plane of interface was obtained using least square plane fitting [20] (also known as the best fit plane). To decide swapping status for oligomeric proteins, the positions of the residues were examined at the interface region of one of the protomers (Here, ‘protomer’ refers to *one* of the chains of a protein engaged in dimer or multimer formation. This is technically different from a ‘monomer’, where there is no evidence of oligomerisation).

Here, we assumed that if a given protein complex is non-domain-swapped then the two protomers will lie completely on opposite sides of the plane. For this purpose, our algorithm uses C^α^ coordinates of each residue to calculate the least-square fit plane. Suppose 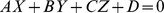 is the equation of least square plane passing through interface residues in a dimer.

If 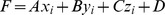


Then,


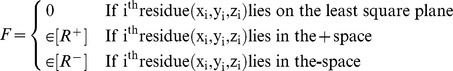


i = i^th^ residue from domain-swapped protomer

(x_i_, y_i_, z_i_) = X,Y and Z coordinates respectively of i^th^ residue C^α^ atom

*F* = Solution of least square plane equation for i^th^ residue C^α^ atom coordinate (x_i_, y_i_, z_i_).


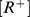
 = Set of all positive real numbers


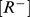
 = Set of all negative real numbers

**Figure 2:**
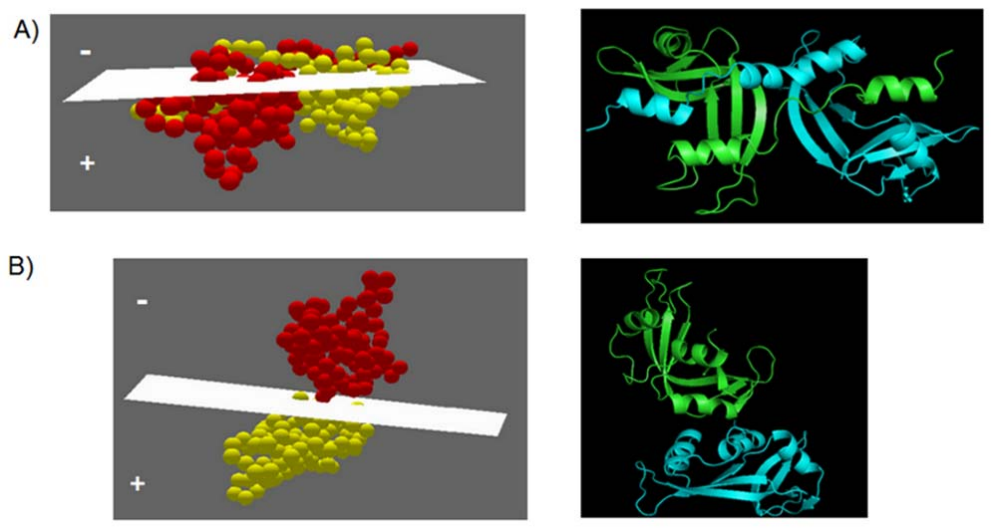
Least Square plane passing through interface residues of domain-swapped form and non-domain-swapped form of ribonuclease. A) Least square plane passing through interface residues of chain A of 1A2W (domain swapped protein). B) Least square plane passing through interface residues of chain A of 1RBB (non-domain-swapped protein and homologue of 1A2W).

“+” space and “−” space are two hypothetical 3D-spaces lying on both sides of least-square plane (Figure 2).

If all C^α^ coordinates of one chain gives all positive or all negative output when substituted in the least square plane equation, then given protein is considered as ‘non-domain-swapped’ structure.

We found that in rare instances, some residues crossed this plane for NDSI. In such cases, we found that the penetrating parts were discontinuous. Therefore, before assigning domain-swapped status to all entries, continuity of swapped domain is checked. In case of complexes with more than two chains, all possible pairs of chains are checked individually. If any pair of chains is found to be domain swapped, then the whole complex will be considered as domain-swapped oligomer.

#### Step 2: Identification of hinge region in domain-swapped structure and structurally equivalent region in non-swapped homologue by successive superposition

**Figure 3:**
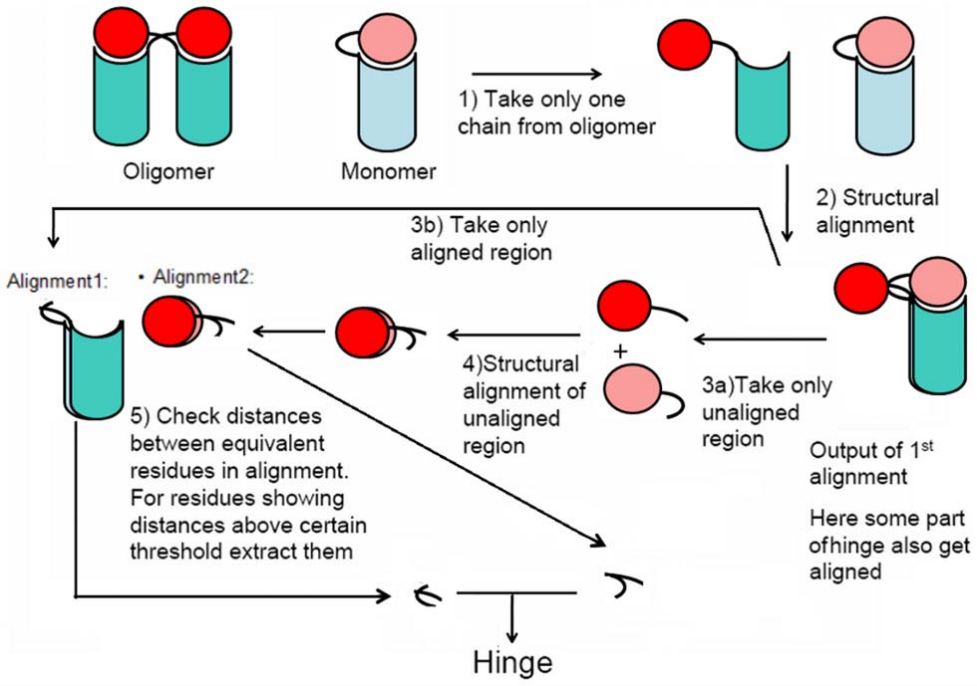
Flowchart of Hinge Identification using structural alignment.

The presence and boundaries of the hinge region can be recognised easily on the graphics for most protein dimers that undergo swapping if structure of non-domain-swapped form is available. However, owing to the large number of domain-swapped entries and to avoid subjectivity for difficult examples, multiple superposition strategies were used as an approach to decide on hinge boundaries. To identify hinge region with good accuracy, first best non-domain-swapped homologue is recognised. Non-domain-swapped homologue of a domain-swapped structure is a closely related protein whose structure is available in Protein Data Bank and is observed not to undergo swapping. The best such homologue is decided based on percent sequence identity with query domain-swapped protein. This algorithm uses two consecutive structure-based superposition and structural alignments between domain-swapped structures and its best non-domain-swapped homologue to identify the hinge region (as shown in **Figure 3**). The method used for superposition is TM-Align [21] structural alignment tool.

The successive superposition and structural alignment of unaligned regions would superpose the swapped region well, leaving behind the hinge region with the highest structural deviation between the domain-swapped and non-domain-swapped protomer. Analysis of both of these structural alignments would show that the consensus equivalent residues would retain all except some part of the hinge region. Aligned residues, lying next to unaligned regions, are also considered part of the hinge region. Where there are continuing uncertainties with the hinge boundaries, we examine the backbone torsion angles to recognise these regions. For example, the structural deviation between hinge regions could be low if there are reverse turns or turns of similar orientations in the hinge-loops. In most of the cases, we observed conformational change of flanking residues from both sides of the identified hinge portion. Therefore, ten flanking residues adjacent to the putative hinge region are further checked for conformational changes by 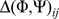 calculation. If 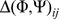 value exceeds 20°, then it is considered as structurally variant position/hinge residue within.


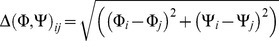


i = Amino acid residue in query chain (domain-swapped protein)

j = Structurally equivalent amino acid residue in non-domain-swapped best homologue


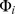
 = φ angle of residue i


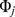
 = φ angle of residue j


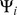
 = ψ angle of residue i in query chain (domain-swapped protein)


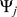
 = ψ angle of residue j in non-domain-swapped best homologue)


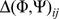
 = Conformational difference between backbone torsional angles of i^th^ and j^th^ residues

The region with continuous conformational change was considered as hinge in the domain-swapped protein and its equivalent region in the non-domain-swapped homologue.

### Structural and sequence analyses of hinge region

Sequence of hinge regions were extracted for protein entries in 3DSwap database. Their non-domain-swapped forms were searched using the HIDE algorithm (as mentioned below in detail). The equivalent region of hinge from non-domain-swapped homologues was extracted and amino acid propensities were calculated for α-helix, β-sheets, loops and hinge regions using following statistical parameters.


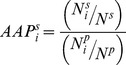



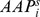
 = Amino acid propensity of amino acid type “i” to be in structure s (s = α-helix, β-sheet, loops or hinge)


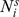
 = Number of amino acid type “i” in structure “s” in dataset


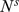
 = Total number residues in structure “s” in dataset


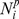
 = Number of amino acid type “i” in whole proteins from dataset


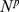
 = Total number residues in whole proteins from dataset

The amino acid propensity at the hinge regions were compared using correlation coefficient against those in other secondary structures.


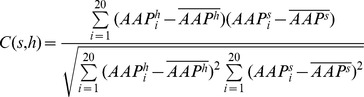



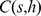
 = Correlation coefficient between amino acid propensity to be in the hinge and that of structure s (s = α-helix, β-sheets or loops)


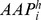
 = Amino acid propensity of type “i” to be in the hinge


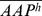
 = Average amino acid propensity to be in the hinge


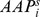
 = Amino acid propensity of type “i” to be in secondary structure “s”


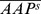
 = Average amino acid propensity to be in secondary structure “s”

The ratio of fraction of hinge and loop contributed were further used to calculate normalized amino acid propensities of the hinge residues.


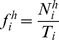



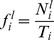



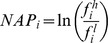



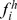
 = Fraction of hinge contributed for amino acid type i


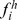
 = Fraction of loop contributed for amino acid type i


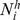
 = Number of residue type i in hinge


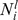
 = Number of residue type i in loop


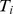
 = Total number of residues of type i in protein


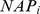
 = Normalized amino acid propensities of the type i hinge residues

(φ, ψ) conformational values of the residues at the hinge region were examined for both domain-swapped oligomer and non-domain-swapped homologues. Srinivasan, et al. 1991 studied conformations of short loops [22]. A similar approach has been used to analyse these hinge regions. Only crystal structures with resolution better than 2 Å were considered for the analysis. Hinge regions with more than five residues long were omitted from the current analysis owing to their conformational heterogeneity. Further, only few observations of hinge regions more than five residues could be observed in the dataset (Figure S1).

### Analysis of interfaces formed in domain-swapped oligomers

Residues were considered as being in the interface only if their solvent accessibility decreases by 1 Å^2^ upon oligomerization.

Following structural features were calculated for each entry at the interface:

1. **Disulphide bonds** [**23**] **.** It is a covalent bond between two cysteine residues, if any i.e. if their C^α^ atoms are within 6.5 Å.
2. **Hydrophobic interactions** [**23]**–[**24**] **.** These are interactions between hydrophobic amino acids (ALA, LEU, ILE, VAL, TRY, TYR, PHE) if their C^β^ atoms are within 7 Å.
3. **Short contacts** [**23]**–[**24**] **.** These are contacts between residues for whose atoms D (function)<0.
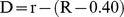

  Where, r = distance between two atoms, R = Sum of Van der Waals radii of those atoms.
4. **Structural Segmentation** [**23**] **.** Interface residues separated by more than five residues were allocated to different segments.
5. **Salt bridge** [**23]**–[**24**] **.** A salt bridge is formed if the side-chain nitrogen and oxygen atoms of two oppositely charged residues are within 4 Å distance.
6. **Protrusion Index** [**23]**–[**25**] **.** Protrusion index for each interface residue was calculated using algorithm proposed by Pintar and coworkers.
7. **Hydrogen bonds** [**23]**–[**24**] **.** Hydrogen bonds were identified using HBOND program implemented in JOY [23] package was used.
8. **Van der Waals interactions** [**23]**–[**24**] **.**

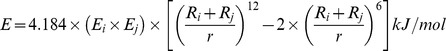

  R = Van der Waals radius for an atom
9. E = Total Van der Waals energy
10. r = distance between the p^ith^ atom and j^th^ atom.
11. (Ramachandran & Sasisekaran, 1968 and Novotny et al., 1997).
12. **Planarity** [**20]**–[**23**] **.** Planarity of each interface surface was measured using least square plane. This plane was calculated as explained in first step of HIDE algorithm. Then RMSD of each interface atom from this plane was calculated. Minor surfaces, having number of interface residues less than four, were ignored.

Interface surface areas of domain swapped interface (DSI) and non-domain swapped interface (NDSI) were calculated using PSA [26] software from JOY 4.3 package [27].

First, all the values for each structural feature were normalized by respective interface surface area. Then paired t-test was performed using R-package to check whether there is any significant difference between the means of the two datasets to be compared.

## Results

### Evaluation of hinge identification algorithm

The performance of the HIDE algorithm (**Table** 1; Tables S1, S2, S3) was evaluated using 104 domain-swapped entries because non-domain-swapped structures were available for these 104 proteins. Hinge regions and their boundaries could be identified by HIDE algorithm for 86 out of 104 entries (around 83% accuracy). For the 18 cases, where non-domain-swapped homologue is available but hinge region could not be identified, the swapped domain coordinates are missing (Table S3). For example, domain swapped structure of human TREM-1 receptor (PDBID: 1Q8M) and its monomeric homologue in mouse (PDBID: 1U9K) could not be compared due to missing N-terminal β-strand in the latter. Where possible, they were extended for detailed structural analyses (parameters consolidated in Table S4).

**Table 1:** Performance of HIDE algorithm for non-redundant entries from 3D-swap database.

Domain swapped queries	Number of entries
Queries for which hinge identified	86
Absence of swapped domain in swapped domain of non-swapped homologue found for queries	18
Non-domain-swapped homologue was not found	78
Total number of queries	182

### Amino acid propensities in hinge region

We observed amino acid propensities for the hinge region in all cases and compared it with propensities of secondary structures (Table 2). We found that propensity values of amino acids to be in the hinge region were negatively correlated with that of alpha helix (−0.50) and beta sheets (−0.38). However, they were positively correlated with loops (0.6), as expected.

**Table 2:** Propensities of amino acids to be in alpha-helix, beta-sheets, loops and hinge.

Amino Acids	Amino acid propensity in alpha helices	Amino acid propensity in Beta sheets	Amino acid propensity in Loops	Amino acid propensity in hinge
A	1.46	0.87	0.71	1
C	0.93	1.37	0.84	0.97
D	0.96	0.54	1.31	0.87
E	1.46	0.65	0.85	0.89
F	1.04	1.44	0.7	0.88
G	0.42	0.36	1.85	0.96
H	0.85	1.16	1.02	0.89
I	0.86	1.85	0.59	0.76
K	1.12	0.86	0.99	1.24
L	1.32	1.15	0.65	0.76
M	1.39	0.76	0.83	0.41
N	0.75	0.66	1.4	1.21
P	0.55	0.35	1.75	1.83
Q	1.25	0.81	0.92	1.1
R	1.17	0.88	0.94	0.86
S	0.68	0.9	1.31	1.41
T	0.78	1.29	0.99	1.02
V	0.84	1.9	0.58	0.82
W	1.13	1.19	0.78	0.86
Y	0.98	1.39	0.78	1.02
Correlation Coefficient	−0.51	−0.38	0.61	1

Amino acids were sorted by their normalized amino acid propensity values and all amino acids obtained the same rank as in loops and in the hinge region. However, correlation between them was not high. We next assumed hinge regions could be special kind of loops and certain amino acids could favour hinge regions over the other. Therefore, we examined the difference in propensities of amino acids in hinge regions and in loops (please see Table 3 for these values in the ascending order). Let us refer to this as ‘normalized propensity value’.

**Table 3:** Normalized amino acid propensities of the hinge residues with reference to amino acids at the loop regions.

Rank	Amino acid	ln(f_h_/f_l_)
1	V	0.34
2	A	0.34
3	Y	0.27
4	I	0.25
5	K	0.22
6	F	0.22
7	Q	0.18
8	L	0.16
9	C	0.15
10	W	0.09
11	S	0.07
12	P	0.05
13	E	0.04
14	T	0.02
15	R	−0.09
16	H	−0.14
17	N	−0.15
18	D	−0.41
19	G	−0.66
20	M	−0.72

A very interesting order was observed, in which Valine and Alanine retained the highest difference in normalized amino acid propensity values, suggesting that these are highly favourable for the hinge region. Glycine and Methionine showed large negative values of difference in propensity values suggesting that those were least favourable amino acids for hinge region, though Glycine has the highest propensity values for loop regions.

There are many experimental studies validating this observation that glycine is least favoured amino-acid in hinge [28]. We also found cases where alanine substitution in the hinge region leads to domain swapping *e.g.* : B1 Domain of Protein L from *Peptostreptococcus magnus* in this protein hinge region is 53–56 and mutation G55A induced domain swapping while same protein with K54G mutation preferred to stay in monomeric form [29]. Likewise, V57N substitution within the hinge region of human cystatin C protein leads to formation of monomer [30]. Interestingly, Valine stands first and asparagine acquires the 17^th^ position according to ranks with respect to difference in normalized-propensity between hinge and loop regions (please see Table 3). Please see Table S5 for additional mutational studies at the hinge regions, where introduction of residues, with higher propensity to occur in hinge region (as in Table 3), induced domain swapping.

However, proline was found to be an exception to this above order *e.g.*: in beta B2-crystallin protein, where 80–88 residue stretch is the hinge region, P80L mutation prevents domain swapping [31], although proline has lower normalized propensity value to be present in the hinge region. On the other hand, the trend is opposite in histone fold protein where deletion of hinge region containing proline induced domain swapping [32]. This anomalous behaviour of proline could be due to backbone conformational switches possible in the presence of this residue (see next section on (φ, ψ) distribution).

### (φ, ψ) distribution of residues in hinge

**Figure 4:**
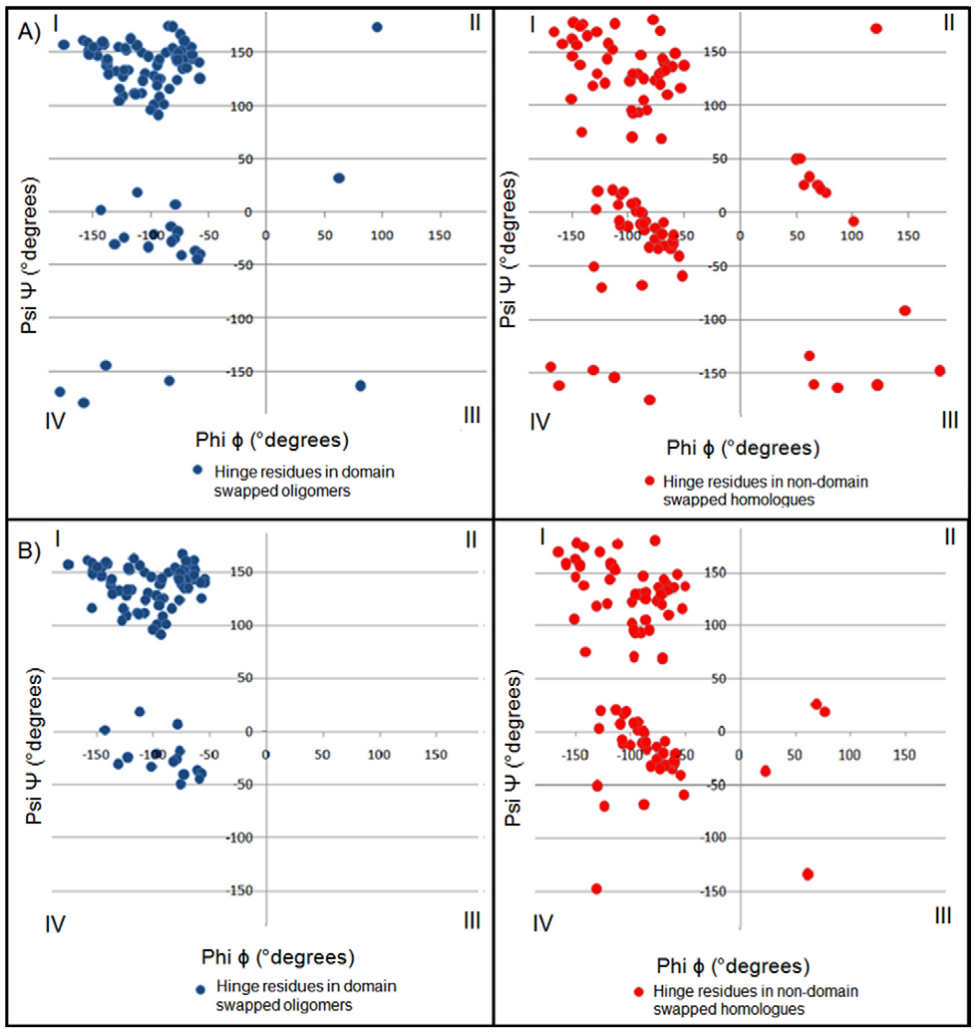
Backbone conformation of hinge regions in domain swapped oligomers. A) (φ,ψ) conformation distribution of all hinge residues. B) (φ,ψ) conformation distribution of non-glycine hinge residues.

**Figure 5:**
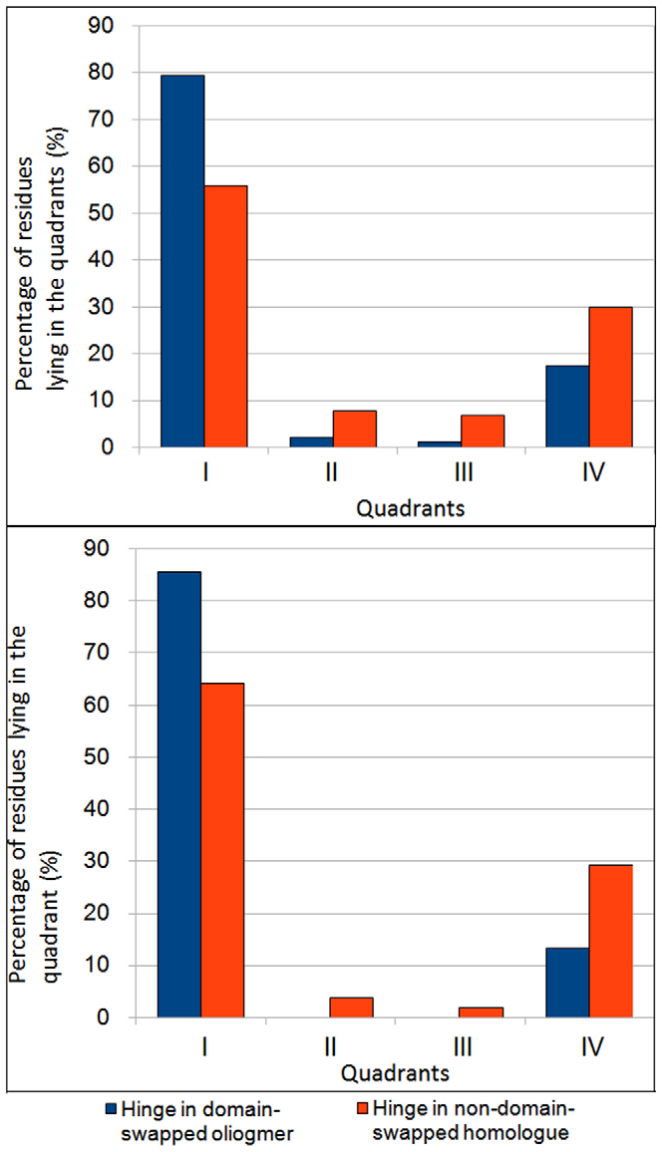
Distribution of backbone conformation of hinge residues where (φ,ψ) is separated into four quadrants (please see **Figure 4** for regions within each quadrant). A) Percent distribution of all hinge residues in all quadrants. B) Percent distribution of non-glycine hinge residues in all quadrants.

The (φ, ψ) distribution of residues in the hinge region of domain-swapped oligomer and its structural equivalent portion from monomeric form was observed (as detailed in Methods) for all amino acid residues (Figure 4A) and for non-glycyl residues (Figure 4B). The calculated percent frequency of all residues and non-glycine residues to be in all four quadrants are shown in Figure 5A and Figure 5B, respectively.

Most of the residues lie in the first and fourth quadrants and few in other two quadrants in domain-swapped examples, whereas a fair portion of monomeric hinge residues, 7.7% and 6.7% lie in the second and third quadrant for monomeric hinge residues, respectively; only observations of 2.2% and 1.1% of residues from oligomeric hinges are observed in second and third quadrants each. Interestingly, most of the hinge regions of oligomeric domain-swapped form were in extended S-shaped form (Figures S2 and S3), e.g. B1 domain in protein L. (φ, ψ) plots of oligomeric and monomeric hinge suggest the same. Interestingly, such S-shaped loops were also observed earlier by Ramakrishnan et al. [33] in proline-rich peptides and shown to form stereochemically feasible “non-β-turn conformations”. Such S-shaped turns are unlike the classical β-turns that could induce sharp reverse turns, providing a structural role for hinge regions in the context of domain swapping.

(φ, ψ) distribution for non-glycine residues of hinge regions from domain-swapped and non-domain-swapped regions remained similar. However, none of the non-glycine residues from oligomeric hinge residues were found in second and third quadrant. But small portion of non-glycine residues i.e. 3.9% and 1.9% from non-domain-swapped form were lying in second and third quadrant, respectively. From (φ, ψ) distribution of non-glycine residues in hinge region in both states, it is clear that higher number of residues in the loop region, equivalent to the hinge region but in the non-domain swapped form, occupy partially allowed region (Figure 4B). Indeed, approximately 3% residues from the monomeric hinge lie in the disallowed region and none from the oligomer lie in the disallowed region (Figure 4B). For example, in the monomeric form of human cystatin-C protein (PDB ID: 3GAX), observation corresponding to Valine57 lies in the disallowed region (in the fourth quadrant with φ and ψ as −130.9°, −147.4° and N-C^α^-C′ angle was 104°). Same residue in the domain-swapped dimer (PDB ID: 1G96) is observed in the first quadrant (−125.9°, 146.2°) in the allowed region and with standard 110° as N-C^α^-C′ angle. Such observations suggest that the domain-swapped form retains lesser stereochemical strain at residues in the hinge region.

**Figure 6:**
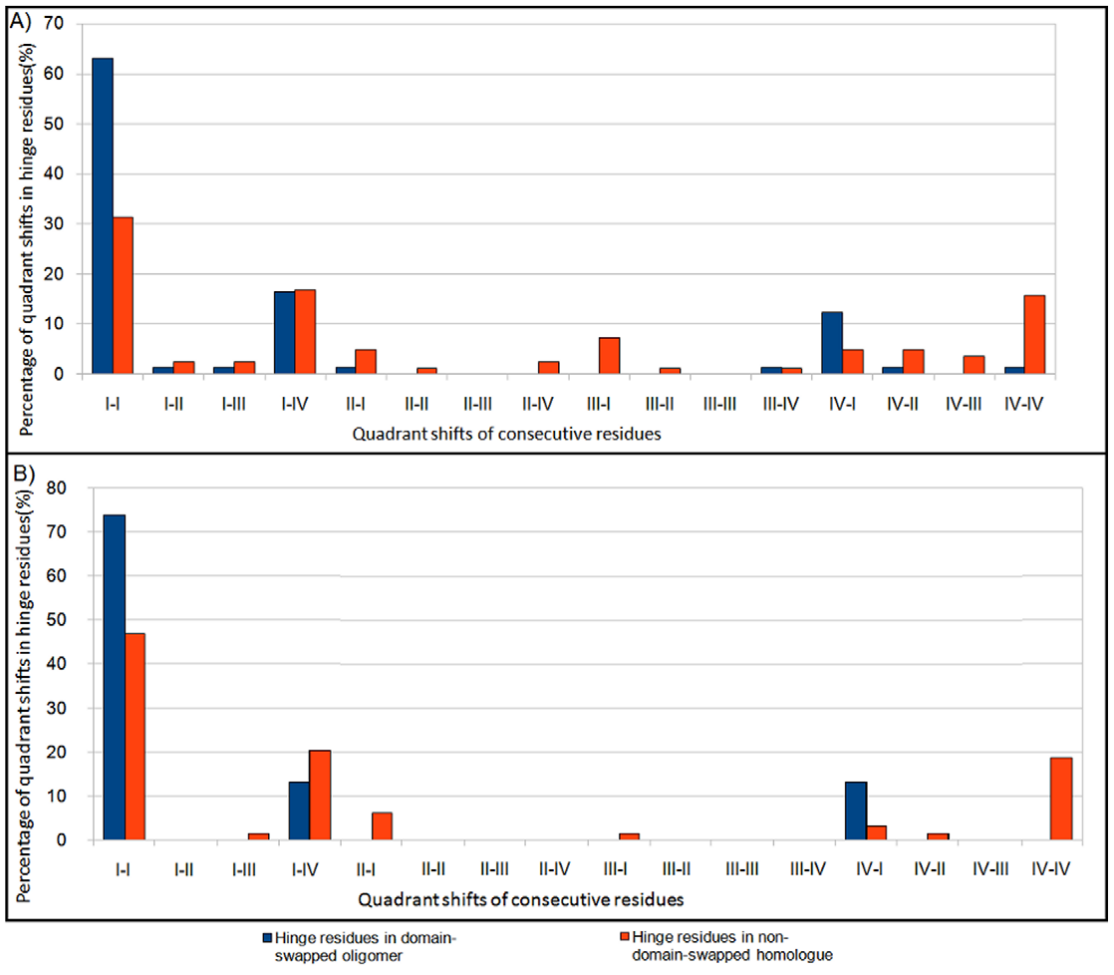
Conformation of residues in hinge region for domain-swapped structure and non-domain swapped homologue. A) Percent distribution of quadrant shifts of all hinge residues. B) Percent distribution of quadrant shifts of non-glycine hinge residues.

**Figure 7:**
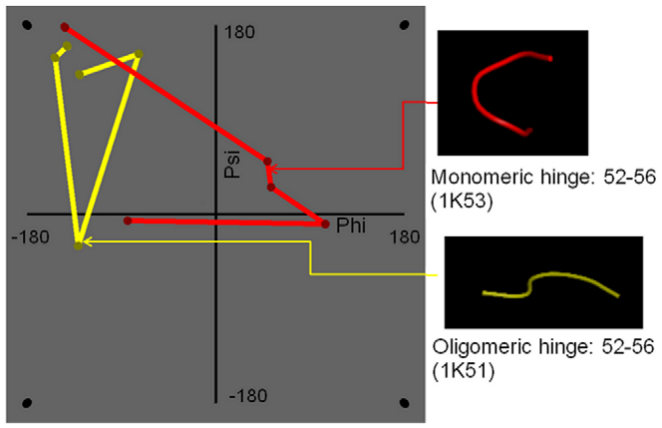
(φ,ψ) conformation of reverse loop monomeric hinge and extended loop oligomeric hinge in protein with inset showing ribbon representation of the actual crystal structures.

**Figure 8:**
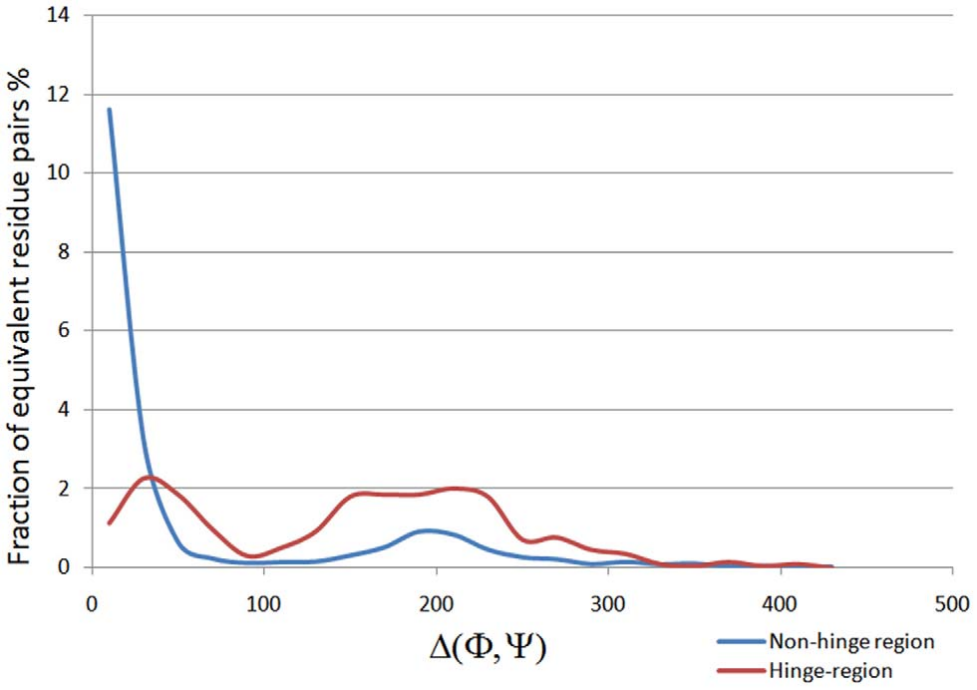
Distribution of 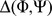
 **values of non-hinge residues and hinge-residues from domain-swapped protomers and their respective non-domain-swapped proteins.**

We performed analysis of (φ, ψ) conformations of adjacent residues in the hinge region and checked their preferences for each quadrant. In domain-swapped proteins, approximately 63% of (φ, ψ) conformations of two consecutive hinge residues lie in the first quadrant, while 31% of that of non-domain-swapped forms lie in the first quadrant (Figures 6 and 7). Significant proportion (17%) of (φ, ψ) conformations of adjacent residues, in the hinge regions of non-domain-swapped homologues, were observed in the fourth quadrant (Figure 6A). Similar trend was observed even after ignoring glycine residues (Figure 6B). In order to eliminate the possibility of these differences arising due to different resolutions of the two proteins under question, we next examined the distribution of 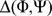 values of hinge residues between non-domain-swapped form and domain-swapped form in comparison with other general loop regions from the same pair of protein structures. The equivalent residues were decided based on structural superposition (Figure 8).

### Structural Analysis

For the study of interfaces in domain-swapped structures, all residues involved in the formation of domain-swapped interface (DSI; identified as detailed in Methods) and domain non-swapped interfaces (NDSI) were chosen for the analysis. Most of the structural features used in this study were shown to be important by Thornton and coworkers [27]. From a non-redundant dataset of 182 entries (Table S1), entries with resolution better than 3.2 Å were alone considered and a total of 171 pairs (Table S2) of DSI and NDSI were compared for these features.

### Comparative analysis of DSI and NDSI

All structural features significantly differ in both the interfaces (see Table S4), except in the number of disulfide bonds. It was observed that both types of interfaces had very few disulfide bonds with no significant difference in their average values. As shown in Table 4 average values of structural features per 1 Å^2^ surface area are placed, along with p-value obtained from paired t-test.

**Table 4:** Structural features with their average values in SI and NSI and p-value generated from paired t-test.

Structural Features	P-value from paired t-test	Average in SI per 1 Å^2^	Average in NSI per 1 Å^2^
Protrusion Index	2.20E-016	1.3351	0.2788
Disulfide bonds	2.86E-001	0.0003	0.0001
Electrostatic interactions	2.20E-016	0.0329	0.0122
Hydrophobic Interactions	2.20E-016	0.0314	0.0070
Salt Bridges	4.70E-013	0.0044	0.0014
Short contacts	2.50E-004	0.0019	0.0004
Structural Segments	8.30E-005	0.0090	0.0067
Van der Waals interactions	2.20E-016	6.0620	1.9227
H-Bonds	2.20E-016	0.0300	0.0099

### Disulfide bonds

Out of 171 proteins, disulfide bonds were present in only ten proteins at DSI, and in five proteins at NDSI. More than 90% of proteins do not possess any disulfide bond in either of the interfaces.

### Salt bridges

In the dataset of 171 proteins, number of salt bridges varies from 0–8 in both DSI and NDSI. On an average, there were four salt bridges per 1000 Å**^2^** in DSI, while one salt-bridge per 1000 Å**^2^** in NDSI. Approximately, four times more salt-bridges are observed in DSI than in NDSI. In 36% and 70% of proteins, salt bridges were absent in DSI and NDSI, respectively. Number of salt bridges correlated well with interface surface area in case of DSI, but no clear correlation found for NDSI. DSI is composed of 24% of charged amino acids, while 21% of NDSI are constituted by charged residues.

### Hydrophobic Interactions

Number of hydrophobic interactions varied from 0–48 in DSI and 0–37 in NDSI. On an average, there were three hydrophobic interactions per 100 Å**^2^** in DSI, while the average hydrophobic interactions are only 0.7 per 100 Å**^2^** in NDSI. It was quite surprising that DSI composed of 51% hydrophobic residues and NDSI composed of 54% hydrophobic residues.

### Structural segmentation

We observed that DSIs were more segmented compared to NDSIs. There were on average 8.81 segments in DSI per 1000 Å**^2^** and 6.67 structural segments per 1000 Å**^2^** for NDSIs.

### Protrusion Index

It was found dramatic differences in the protrusion index for DSI and NDSI. The average protrusion index per residue was 1.30 for residues in DSI and 0.27 for residues in NDSI. This suggests side-chains of residues in DSI were approximately five times more protruded outside compared to residues in NDSI.

### Short Contacts

There were 3.5 times more short contacts in DSI compared to NDSI. On an average, there were 3.5 short contacts per 1000 Å**^2^** in DSI, while approximately 1 per 1000 Å**^2^** in NDSI.

### Van der Waals interactions

In DSI, Van der Waals interactions were more than NDSI. On an average, there were 6.71 interactions per 1 A sq in DSI and 2.16 per 1 Å**^2^** in NDSI.

### Hydrogen bonds

DSI contains three times more hydrogen bonds than that of NDSI. There were three hydrogen bonds per 100 Å^2^ while one per 100 Å^2^ sq was observed in NDSI.

### Planarity

Surprisingly, we found that NDSI was more planar compared to DSI. But we found that numbers of residues in NDSI were very less compared to DSI. Around 45.6% cases were having less than or equal to three interface residues. While only three cases were found to contain less than three residues in DSI. On an average, there were 12 residues in NDSI, while 43 residues in DSI. This might be one reason of having very less RMSD i.e. more planarity for NDSI.

## Discussion

### Geometric Algorithm to identify hinge boundaries starting from domain-swapped and non-domain-swapped pairs

We have developed a structure-based algorithm that uses plane analytical geometry, to identify domain-swapped oligomers and non-domain-swapped proteins automatically with high sensitivity and good specificity. Using the best non-domain-swapped homologue, residues with large conformational variations can be identified easily. Based on this criterion, it is possible to prioritize residues critical for domain swapping for further studies. This algorithm can also suggest pairs of domain-swapped and non-domain-swapped homologous structures which can be used for further comparative structural and sequence analysis which, in turn, may provide further insight into the mechanism of domain swapping.

### Sequence propensity of amino acids in swapped interface and hinge

A clear understanding of amino acid composition of swapped interface and preferred amino acids is important for domain swapping mechanism [34]. Our comparison of amino acid propensities, along with compositional studies of interface residues, may help in identifying favourable and unfavourable residues for domain swapping. Such analysis may further help to decide boundaries of hinge region and importance of residues in hinge regions.

Clear difference between amino acid propensities were observed between their preference to be in the loop and hinge regions. Interestingly, such trends were found to be in agreement with experimental data derived from independent approaches. The higher rank amino acids were very favourable to be in hinge region and several mutational studies (such as random mutations) showed that if lower ranked amino acid were substituted by higher rank amino acids, it leads to domain swapping except for proline. Proline favours extended form of loop which is favourable to form domain-swapped oligomers [33]. These differences in propensities can be used to predict hinge from structure of monomeric form of protein. In combination with other parameters, it may be possible to predict hinge from mere protein sequence information.

### Analysis of backbone conformation of hinge region

A (φ, ψ) conformational analysis clearly indicated that oligomeric hinge regions were sterically more optimized than that monomeric hinge. Though glycine residues were also present in oligomeric hinge, they preferred to stay in first and second quadrant, unlike glycine residues in the monomeric hinge (Figure 5).

Around 86% of non-glycine residues were lying in first quadrant confirming (Figure 8) that oligomeric hinge prefers extended conformation. Some non-glycine hinge residues from nonswapped form were also lying in region for alpha-L and alpha-R (Figure 6), while none of the non-glycine residues from oligomeric hinge were present in those regions, perhaps to form optimum intramolecular interactions. In domain-swapped oligomers, these steric hindrances have been relaxed while preserving these intramolecular interactions in form intermolecular interactions. This steric hindrance can be a driving force for oligomerization by domain swapping.

From experimental studies [35], it was shown that the presence of proline in hinge region induces domain swapping. Ramakrishnan et al. [33] observed that proline containing loops adopt extended S-shape non-β turns. Remarkably, a similar extended S-shape was observed in structural alignment of the hinge regions from domain swapped oligomers (Figure S1, Figure 7), while reverse loop conformation was observed in case of monomeric hinge regions (Figure S2).

### Structural features

Intramolecular interactions were significantly different in DSI compared to NDSI, except for the disulfide bonds. Both the interfaces had very few disulfide bonds and most of the entries lacked disulfide bonds in both interfaces. One probable reason for this might be the partial unfolding step. In domain swapping mechanism, the first partial unfolding is required in order to form domain-swapped structure. Partial unfolding to form open monomer starts at DSI. If DSI contains any disulfide bonds, then this partial unfolding will require high energy to break disulfide bonds and subsequently increasing energy barrier between monomeric and oligomeric form.

DSI is present in monomeric form as well as in oligomeric form. Hence, this interface is evolved to optimize energy of the structure by selecting favourable interactions. Hence all favourable interactions viz. Electrostatic interactions, salt bridges, hydrogen bonds, hydrophobic interactions and Van der Waals interactions, short contacts were significantly more in DSI than that in NDSI.

One more possible reason for hydrophobic interactions to be more in DSI might be size of DSI. As average surface area of DSI is approximately two times more than that of NDSI, it contains larger core-interface region, where hydrophobic interactions are more prevalent.

It is quite obvious to have high protrusion index values for residues in DSI compared to NDSI after finding that inter-chain hydrophobic interactions and salt bridges were high in number in DSI. To form salt bridges or to make hydrophobic interactions, side chains have to adopt rotameric form which permits side chain atoms to protrude out towards the interacting chain. These structural features analysis might help us to predict DSI and possible NDSI from given monomeric form alone.

Sequence and structural analyses have been performed on the hinge and interface of domain-swapped entries. In the process, we have developed a structural algorithm, HIDE, which can identify non-domain swapped homologues of given protein and the boundaries of hinge region. This tool can be further useful to study evolutionary relationships as well. This algorithm also tells about most important residue for domain-swapping event which can be a potential drug target in proteins involved in amyloid formation and related to diseases like Alzheimer's disease.

The propensity and relative order of preference of amino acids to be in hinge regions during domain swapping may be very helpful in predicting hinge from protein sequence. This order can also provide clues as to which substitution mutation in hinge region of given protein possibly induces domain swapping in it.

Structural characterization of DSI and NDSI can provide clues to understand quasi-domain swapping i.e. why this mechanism is observed in some proteins and not in the others. The thorough analysis of hinge residues will be useful for researchers to understand domain swapping mechanism. All these observations can be used further to find potential candidates for domain swapping from monomeric structure alone.

## Supporting Information

Figure S1 Hinge length distribution in our dataset.
(TIF)

Figure S2 Structural alignment of oligomeric hinge regions with three flanking residues.
(TIF)

Figure S3 Structural alignment of monomeric hinge regions with three flanking residues.
(TIF)

Table S1 Dataset used to query HIDE algorithm.
(XLS)

Table S2 Performance of HIDE server for each query.
(XLS)

Table S3 Number of interactions in DSI and NDSI per 1 Å2 for non-redundant 3DSwap database.
(XLS)

Table S4 Dataset used for structural analysis.
(XLS)

Table S5 Additional cases supporting derived order of amino-acid preference in hinge region.
(DOC)
