# Supplementary figures and images for: Analysis of Domain-Swapped Oligomers Reveals Local Sequence Preferences and Structural Imprints at the Linker Regions and Swapped Interfaces
Source: PLoS One. 2012 Jul 27;7(7):e39305. doi: 10.1371/journal.pone.0039305 (PMC3407178; doi:10.1371/journal.pone.0039305)

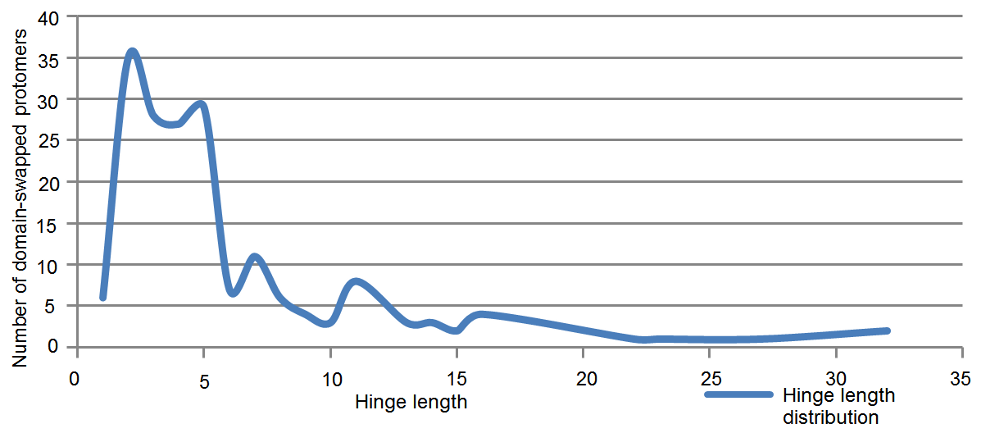

Supplement: Figure S1 — Hinge length distribution in our dataset. (TIF) [file pone.0039305.s001.tif]

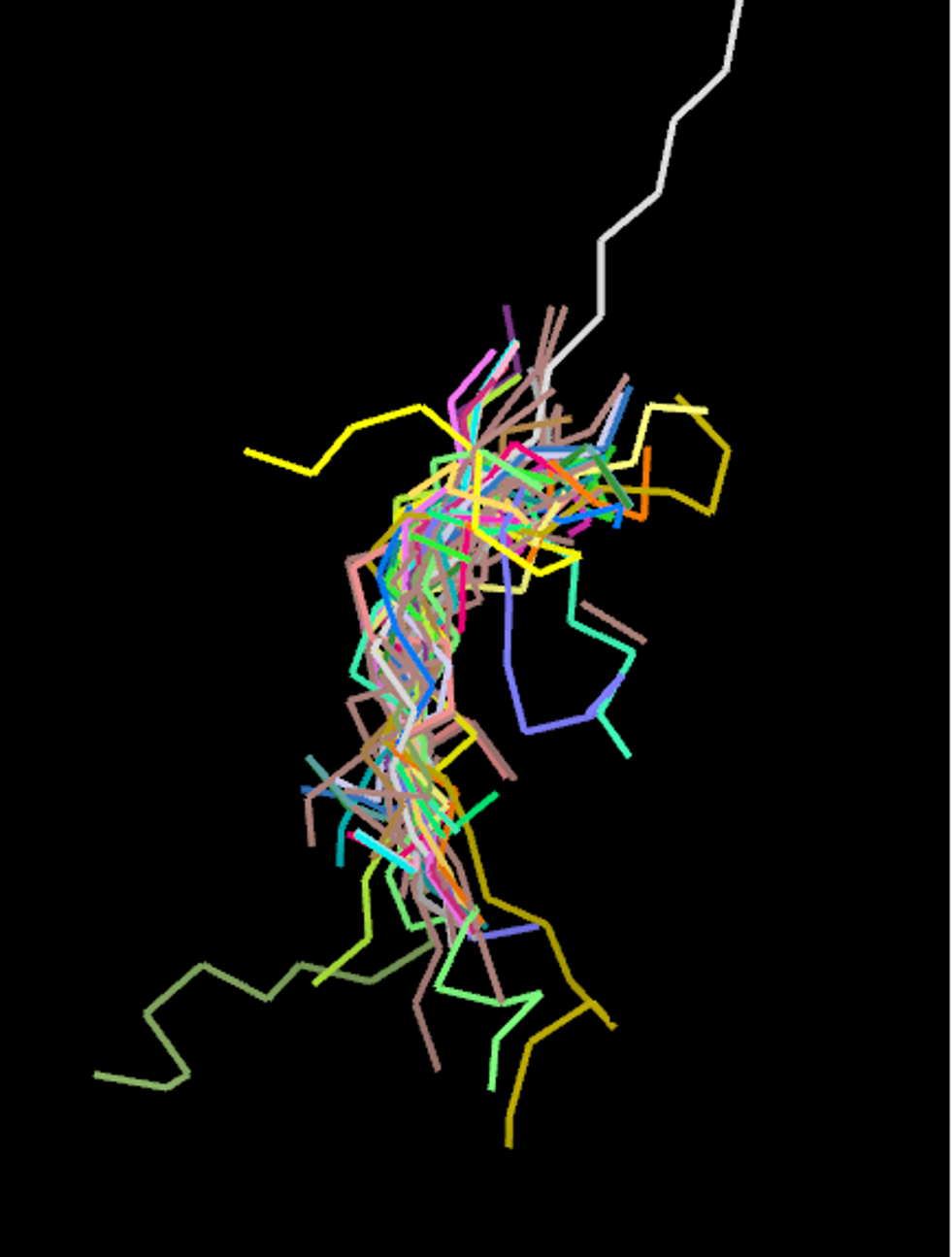

Supplement: Figure S2 — Structural alignment of oligomeric hinge regions with three flanking residues. (TIF) [file pone.0039305.s002.tif]

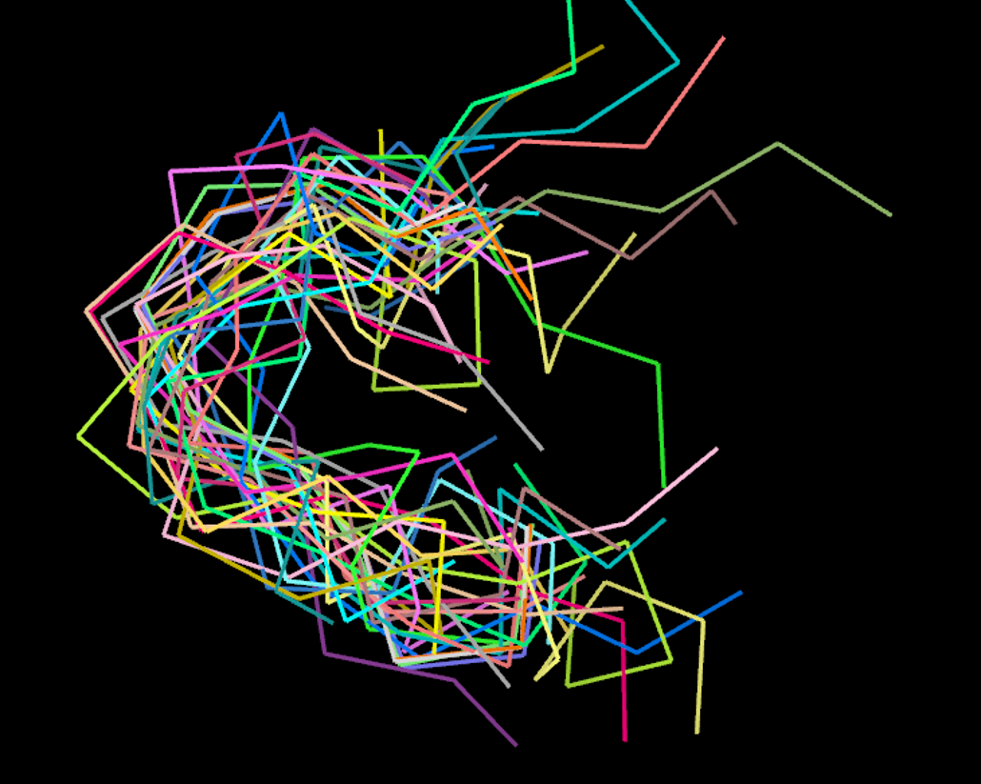

Supplement: Figure S3 — Structural alignment of monomeric hinge regions with three flanking residues. (TIF) [file pone.0039305.s003.tif]
