# Supplementary material for: Analysis of Domain-Swapped Oligomers Reveals Local Sequence Preferences and Structural Imprints at the Linker Regions and Swapped Interfaces
Source: PLoS One. 2012 Jul 27;7(7):e39305. doi: 10.1371/journal.pone.0039305 (PMC3407178; doi:10.1371/journal.pone.0039305)
Supplement: Table S5 — Additional cases supporting derived order of amino-acid preference in hinge region. (DOC) [file pone.0039305.s008.doc]

**Table S5: Additional cases supporting derived order of amino-acid preference in hinge region.**

| **Protein name** | **Wild type protein** | | | **Mutant protein** | | | |
| --- | --- | --- | --- | --- | --- | --- | --- |
|  | **Amino acid at hinge** | **Rank** | **Domain swapping status** | **Amino acid at hinge** | **Rank** | **Domain swapping status** | **Type of mutation** |
| Cystatin C^[S1]^ | Val (57) | 2 | Domain swapped | D | 18 | Non domain swapped | Substitution |
| Cystatin C^[S1]^ | Val (57) | 2 | Domain swapped | N | 17 | Non domain swapped | Substitution |
| protein L^[S2]^ | Gly (55) | 19 | Non domain swapped | A | 1 | Domain swapped | Substitution |
| protein L^[S2]^ | Lys (54) | 5 | Domain swapped | Gly | 19 | Non domain swapped | Substitution |
| Cystatin C^[S3]^ | - | - | Non domain swapped | Q | 7 | Domain swapped | Insertion |
| Cyanovirin N^[S4]^ | Pro(51) | 12 | Domain swapped | Gly | 19 | Non domain swapped | Substitution |
| human cystatin C^[S5]^ | Leu | 8 | Non domain swapped | Gln | 7 | Domain swapped | Substitution |
| SgrAI^[S6]^ | Pro(27) | 12 | Domain swapped | Gly | 19 | Non domain swapped | Substitution |

S1) Aneta Szymańska, Adrianna Radulska, Paulina Czaplewska, Anders Grubb, Zbigniew Grzonka and Sylwia Rodziewicz-Motowidło. (2009) Governing the monomer-dimer ratio of human cystatin c by single amino acid substitution in the hinge region. *Acta Biochimica Polonica* 56(3/2009): 455–463.

S2) Brian Kuhlman, Jason W. O’Neill, David E. Kim, Kam Y. J. Zhang‡, and David Baker. (2001) Conversion of monomeric protein L to an obligate dimer by computational protein design. *PNAS* 98(19): 10687–10691

S3) Olafson I., Grubb A. (2000) Hereditary cystatin C amyloid angiopathy. Amyloid 7: 70-79.

S4) Laura G. Barrientos, John M. Louis, Istvan Botos, Toshiyuki Mori, Zhaozhong Han, Barry R. O’Keefe, Michael R. Boyd, Alexander Wlodawer and Angela M. Gronenborn (2002) The domain-Swapped dimer of cyanovirin-N is in a metastable folded state: Reconciliation of X-Ray and NMR structures. *Structure* 10: 673–686.

S5) Andrew D. Miranker (2005) Fibres hinge on swapped domains. *Nature Structural Biology* 437:197-198

S6) Park CK, Joshi HK, Agrawal A, Ghare MI, Little EJ, et al. (2010) Domain Swapping in Allosteric Modulation of DNA Specificity. PLoS Biol 8(12): e1000554
